# *Trueperella pyogenes* and* Brucella abortus* Coinfection in a Dog and a Cat on a Dairy Farm in Egypt with Recurrent Cases of Mastitis and Abortion

**DOI:** 10.1155/2018/2056436

**Published:** 2018-03-20

**Authors:** Gamal Wareth, Mohamed El-Diasty, Falk Melzer, Jayaseelan Murugaiyan, Amir Abdulmawjood, Lisa D. Sprague, Heinrich Neubauer

**Affiliations:** ^1^Friedrich-Loeffler-Institut, Federal Research Institute for Animal Health, Institute of Bacterial Infections and Zoonoses, Naumburger Str. 96a, 07743 Jena, Germany; ^2^Department of Pathology, Faculty of Veterinary Medicine, Benha University, Moshtohor, Toukh 13736, Egypt; ^3^Animal Health Research Institute-Mansoura Provincial Laboratory, Mansoura, Egypt; ^4^Institute of Animal Hygiene and Environmental Health, Centre for Infectious Medicine, Freie Universität Berlin, Robert-von-Ostertag-Str. 7-13, 14163 Berlin, Germany; ^5^Institute of Food Quality and Food Safety, Research Center for Emerging Infections and Zoonoses, University of Veterinary Medicine Hannover, Bünteweg 17, 30559 Hannover, Germany

## Abstract

*Trueperella pyogenes* was isolated from a dog and a cat with a mixed infection with* Brucella abortus*. Both lived on a dairy cattle farm with a history of regular cases of abortion and mastitis. Identification of the bacteria was done by means of MALDI-TOF MS, loop-mediated isothermal amplification (LAMP) based on* cpn*60, partial 16S rRNA sequencing, and growth on Loeffler Serum Medium. Isolation of* Trueperella pyogenes* on the dairy farm highlights its neglected role in reproduction failure and draws attention to its effects in the dairy industry in Egypt. Diagnosis and control of abortion in Egypt should include* Trueperella pyogenes *as one of possible causes of abortion.

## 1. Introduction

*Trueperella (T.) pyogenes* is Gram positive, haemolytic, nonmotile, non-spore-forming, facultative anaerobic coccobacillus. The species was originally known as* Corynebacterium pyogenes* and* Actinomyces pyogenes* and then as* Arcanobacterium pyogenes*. Based on the 16S rRNA signature nucleotide comparisons and menaquinone and phospholipid compositions, the genus was divided into* Arcanobacterium* and* Trueperella* gen. nov., in honour of the German microbiologist Hans G. Trüper. Currently, the genus* Trueperella *encompasses five different species, which are,* T. pyogenes, T. abortisuis, T. bonsai, T. bernardiae*, and* T. bialowiezensis *[1].

*Trueperella pyogenes* is a commensal and an opportunistic pathogen which can cause a variety of suppurative infections in livestock and wildlife and can elicit sporadic cases of abortion due to endometritis and placentitis at any stage of gestation [2]. The bacteria can affect a wide range of animal species, including cattle, camel, horse, swine, antelope, bison, chicken, pigeon, turkey, deer, elephant, gazelle, macaw, and reindeer [3], as well as companion animals such as dogs and cats [4].* T. pyogenes* has recently been isolated from lung abscesses of slaughtered one-humped camels in Cairo presenting with clinical and subclinical pulmonary infection [5]. Different suppurative* T. pyogenes* infections in livestock and companion animals are associated with a variety of virulence factors, particularly exotoxin pyolysin and adhesins (fimbriae, neuraminidases, and collagen-binding protein) [6].

The present study describes the isolation of* T. pyogenes* in the uterine discharge of a bitch after abortion and from a cat presenting with pyometra. Both animals lived on a dairy farm with recurrent cases of abortion and mastitis due to* Brucella (B.) abortus *biovar (bv) 1 infections [7]. The aim of the current study was to identify and highlight a neglected pathogen in Egypt which can circulate in pet animals in contact with livestock and cause reproductive failure within the dairy industry.

## 2. Material and Methods

### 2.1. Bacteriology

Uterine discharges were collected from the bitch and the cat under sterile conditions after positive* Brucella* serology using Rose Bengal Test and Complement Fixation Test and sent to the OIE reference laboratory of brucellosis at the Friedrich-Loeffler-Institut for* Brucella* identification and biotyping. During cultivation on blood agar for* Brucella* biotyping, two types of bacterial colonies were found. Identification and biotyping of the putative* Brucella* isolates were done by assessing colony morphology, biochemical reactions, CO_2_ requirement, production of H_2_S, growth in the presence of the dyes thionine and fuchsine, reaction with monospecific antisera (A, M, and R), and phage lysis (Wb, Tb, and F25) [8]. Putative* Trueperella* strains were additionally grown on Loeffler Serum Medium (Himeda, Mumbai, India) and tested for catalase activity.

### 2.2. MALDI Based Species Identification

The microbial species identification was carried out using matrix-assisted laser desorption/ionization (MALDI-TOF MS) as described elsewhere [9]. In brief, bacteria from single colonies were inactivated by the addition of 300 *μ*L of HPLC grade water and 900 *μ*L of absolute ethanol. For protein extraction, the suspensions were centrifuged at 11290*g* for 2 min, the supernatants discarded, and the resulting cell pellets air-dried to remove traces of ethanol. Each pellet was reconstituted in 50 *μ*L of 70% formic acid and 50 *μ*L of acetonitrile. The samples were then sonicated (100% amplitude and 1.0 duty cycle) for 1 min on ice using a sonicator (UP100H; Hielscher Ultrasound Technology, Teltow, Germany). Next, samples were centrifuged at 11290*g* for 5 min at room temperature and the clear supernatant was collected. One *μ*L of each supernatant was spotted onto the MALDI target (MSP 96 target polished steel (MicroScout Target) plate; Bruker Daltonik, Bremen, Germany), air-dried, and overlaid with 1.0 *μ*L of saturated *α*-cyano-4-hydroxycinnamic acid matrix solution (in 50% acetonitrile and 0.25% trifluoroacetic acid). The MALDI measurements were carried out using a Microflex LT (Bruker Daltonics, Bremen, Germany) instrument and MBT Compass Explorer 4.1 software (Bruker Daltonics, Bremen, Germany). The MALDI Biotyper manufacturer's recommendation on log score value of 0–3 for species identification was followed. Score values between 2.3 and 3.0 indicate “highly probable species identification”; values between 2.0 and 2.29 indicate a “secure genus identification and probable species identification”; values between 1.7 and 1.99 indicate “probable genus identification”; and values between 0 and 1.69 indicate “no reliable identification.”

### 2.3. DNA Extraction, AMOS-PCR, and Partial 16S rRNA Sequencing

Genomic DNA was extracted from heat inactivated individual bacterial colonies using the High Pure PCR template preparation kit (Roche Applied Sciences, Mannheim, Germany) according to the manufacturer's instructions. AMOS-PCR for* B. abortus, B. melitensis, B. ovis*, and* B. suis* was done as previously described [7, 10]. Partial 16S rRNA genes of the bacterial isolates were amplified by PCR with the 16SUNI-L (5′-AGA GTT TGA TCA TGG CTC AG-3′) and 16SUNI-R (5′-GTG TGA CGG GCG GTG TGT AC-3′) primer pair (Jena Bioscience GmbH, Jena, Germany) to generate amplicons of approx. 1,400-bp [11]. PCR products were analyzed by agarose gel electrophoresis, bands were cut out, and DNA was purified using a Gel Extraction Kit (Qiagen, Hilden, Germany) according to the manufacturer's recommendations. Cycle sequencing of the partial 16S rRNA genes was done in both directions by using forward and reverse amplification primers with a BigDye Terminator Version 1.1 Cycle Sequencing Kit (Applied Biosystems, Darmstadt, Germany) according to the recommendations of the manufacturer. Sequencing products were analyzed with an ABI Prism 3130 Genetic Analyzer (Applied Biosystems). Identification of isolates was done by a BLAST search (https://www.ncbi.nlm.nih.gov/blast/) using 16S rRNA gene sequences.

### 2.4. Loop-Mediated Isothermal Amplification (LAMP)

A newly designed loop-mediated isothermal amplification (LAMP) assay based on* cpn60* (encoding for chaperonin 60) was carried out in a total volume of 25 *μ*L containing 0.5 *μ*L each of pho-F3 and Pyo-B3 primer (25 pmol/*μ*L) equivalent to 0.5 *μ*M final concentration, 1 *μ*L each of Pyo-LoopF and Pyo-LoopB primer (25 pmol/*μ*L) equivalent to 1.0 *μ*M final concentration, 2 *μ*L each of Pyo-FIP and Pyo-BIP primer (25 pmol/*μ*L) equivalent to 2.0 *μ*M final concentration, and 15 *μ*L Isothermal Master Mix Iso-001 (Optigene, Horsham, UK). Subsequently, 3 *μ*L DNA was added as a template. The LAMP assay was run at 70°C for 30 min with a melting curve analysis step (annealing curve 98°C to 80°C ramping at 0.05°C per s) in a portable real-time fluorometer (Genie II®, Optigene, West Sussex, UK) according to the manufacturer's instructions as previously described [12].

## 3. Results and Discussion

**Figure 1:**
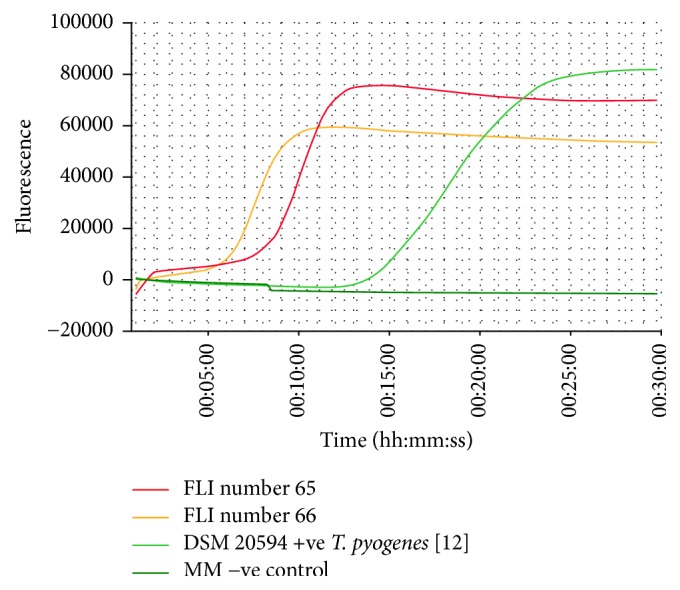
Loop amplification signal of the LAMP products of the two* T. pyogenes* isolates, the reference strain* T. pyogenes *DSM 20594, and the negative control.

**Figure 2:**
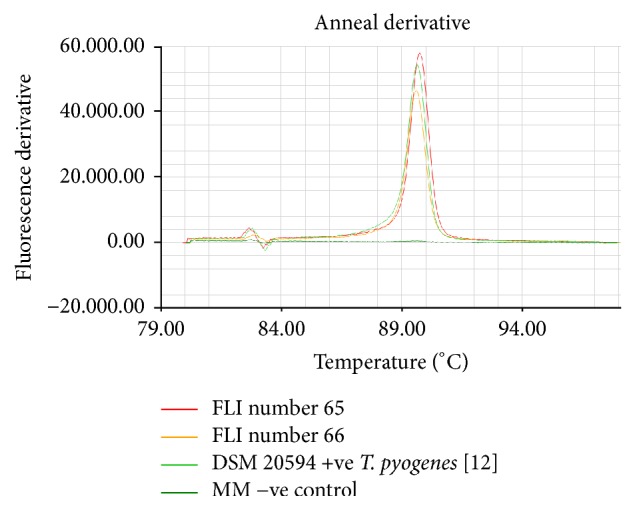
Annealing reaction curves of the respective amplicons.

In addition to* B. abortus* bv1, a Gram positive, catalase negative, aerobic, nonmotile, ß-haemolytic bacterium was recovered from a bitch who had recently aborted and from a cat suffering from an open pyometra. Both animals lived on a dairy farm with regular cases of abortion and mastitis [7]. These recovered bacteria produced H_2_S and were able to grow under aerobic conditions with and without CO_2_. They did not react with the tested monospecific antisera and phages specific for* Brucella* and produced no amplicon in the AMOS-PCR. MALDI-TOF MS identified these bacteria as* T. pyogenes* with a log score of 2.18 (dog isolate) and a log score of 2.02 (cat isolate), respectively. These log score values confirm the identification at the species level. MALDI-TOF MS for* Trueperella* differentiation with a log score around 2 shows comparable discriminating power with molecular methods; it is a rapid and accurate tool for* T. pyogenes* diagnosis [13]. Partial 16S rRNA sequencing confirmed the isolates to be* T. pyogenes* and cultivation on Loeffler serum revealed the typical pitting of the serum slope. The species was also identified using the* cpn60* LAMP assay. A loop amplification signal of the LAMP products was observed for both* T. pyogenes* isolates and for the reference strain* T. pyogenes *DSM 20594 and none in the negative control containing water and LAMP-Mastermix (Figure 1). The mean of the annealing temperature of the amplicons was 89,6°C sd ± 0.05 s (Figure 2). The* cpn60* LAMP assay allowed a reliable, rapid, and low cost identification of* T. pyogenes* (Table 1).

The chaperonin 60 encoding gene has been previously used for the identification of various Gram positive bacteria and a chaperonin sequence database containing a large collections of sequences including gene* cpn60* of* T. pyogenes* is available. This was comparable to the previously described LAMP-mediated identification of* A. pluranimalium* using* pla* gene [14].

*Trueperella pyogenes* is a ubiquitous occurring organism and is frequently found as a commensal in the oropharynx, upper respiratory tract, and gastrointestinal tract of livestock [2]. However, underlying chronic illness, innate immunity, poor animal husbandry, and production methods appear to influence the virulence of the agent [15–18]. It can readily be transmitted by biting flies and contaminated farm and dairy equipment [6].

In Egypt, the dairy industry suffers from large financial losses due to reduced fertility and milk yield as a consequence of uterine infections and mastitis caused not only by brucellosis [5] but also by other agents such as* E. coli*,* Fusobacterium necrophorum*, and* T. pyogenes* [19, 20]. Although* T. pyogenes* is a well-known agent causing reproductive disorders in male and female livestock species [4], its role is neglected as primary cause of abortion in cattle, as diagnosis of abortion is focusing only on predominately classical agents, such as* Brucella* species. Circulating of the bacterium in dog and cat kept in dairy farm with history of abortion and mastitis is alarming and representing a potential reservoir of infection for livestock. Thus screening for this agent should be considered in cases of seronegativity to* Brucella*. On the other hand, eradication of pet animals has to be implemented and taken into account in control measures to reduce dissemination of pathogens.* T. pyogenesis *is not a sufficiently known pathogen in animals and humans because of inadequate identification of this bacterium that should be better known to clinical microbiologists. Diagnosis of abortion in cattle and control has to include* T. pyogenesis *especially in farm suffering from mastitis and sporadic abortion.

## Figures and Tables

**Table 1:** Results of LAMP including detection time and annealing temperature of each isolate and positive and negative control.

Number	Isolate number 1	Isolate number 2	Positive control	Negative control
Sample ID	15RB7429H	15RB7430H	*T. pyogenes* DSM 20594	HPLC water and Master mix
Result	+ve	+ve	+ve	−ve
Detection time	10:00	7:30	17:30	00
Annealing	89.7	89.6	89.6	00
